# A calcified amorphous tumor that developed on both sides of the atrioventricular valve annulus

**DOI:** 10.1007/s12574-015-0267-z

**Published:** 2015-11-19

**Authors:** Masaki Kinoshita, Hideki Okayama, Go Kawamura, Tatsuya Shigematsu, Tatsunori Takahashi, Toru Miyoshi, Akinori Higaki, Kayo Hara, Yoshitaka Kawata, Go Hiasa, Tadakatsu Yamada, Yukio Kazatani, Yutaka Hayashi

**Affiliations:** Department of Cardiology, Ehime Prefectural Central Hospital, 83 Kasugamachi, Matsuyama, Ehime 790-0024 Japan; Department of Cardiology, South Matsuyama Hospital, Matsuyama, Japan

**Keywords:** Calcified amorphous tumor, Transthoracic echocardiography, Transesophageal echocardiography, Hemodialysis, Heart failure

## Abstract

We report a rare case of a hemodialysis patient with a calcified amorphous tumor (CAT) on both sides of the atrioventricular valve annulus. A 70-year-old female who had received hemodialysis for 23 years because of chronic glomerulonephritis presented to our hospital with acute heart failure. Echocardiography indicated the presence of mobile cardiac masses on the mitral valve and tricuspid valve annulus. We suspected the presence of a cardiac tumor or vegetation. The patient received 3 g/day sulbactam-ampicillin and 60 mg/day gentamicin. Surgery was performed on the 14th day after hospital admission. The patient underwent mitral valve replacement, tricuspid annuloplasty, and tumor resection. Based on the pathological findings, the cardiac tumor was diagnosed as a CAT.

## Case

**Fig. 1:**
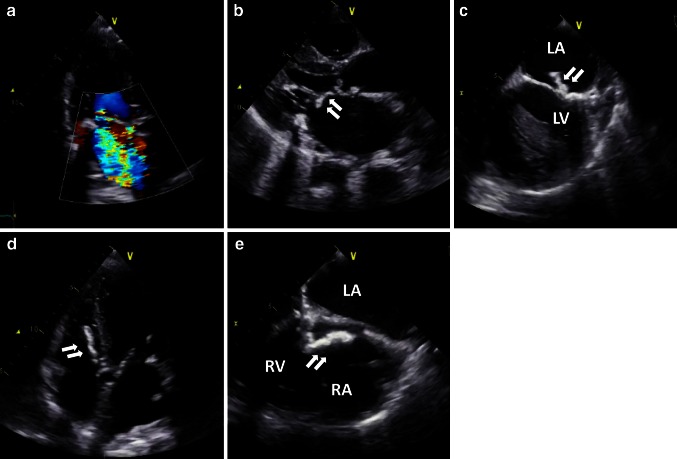
Transthoracic echocardiography (TTE): color doppler image showing severe mitral regurgitation (**a**), parasternal long-axis (**b**) and apical four-chamber view (**d**). Transesophageal echocardiography (TEE): parasternal long-axis (**c**) and apical four-chamber view (**e**). *RA* right atrium; *RV* right ventricle; *LA* left atrium; *LV* left ventricle. *White arrows* indicate a mobile, highly echoic mass

**Fig. 2:**
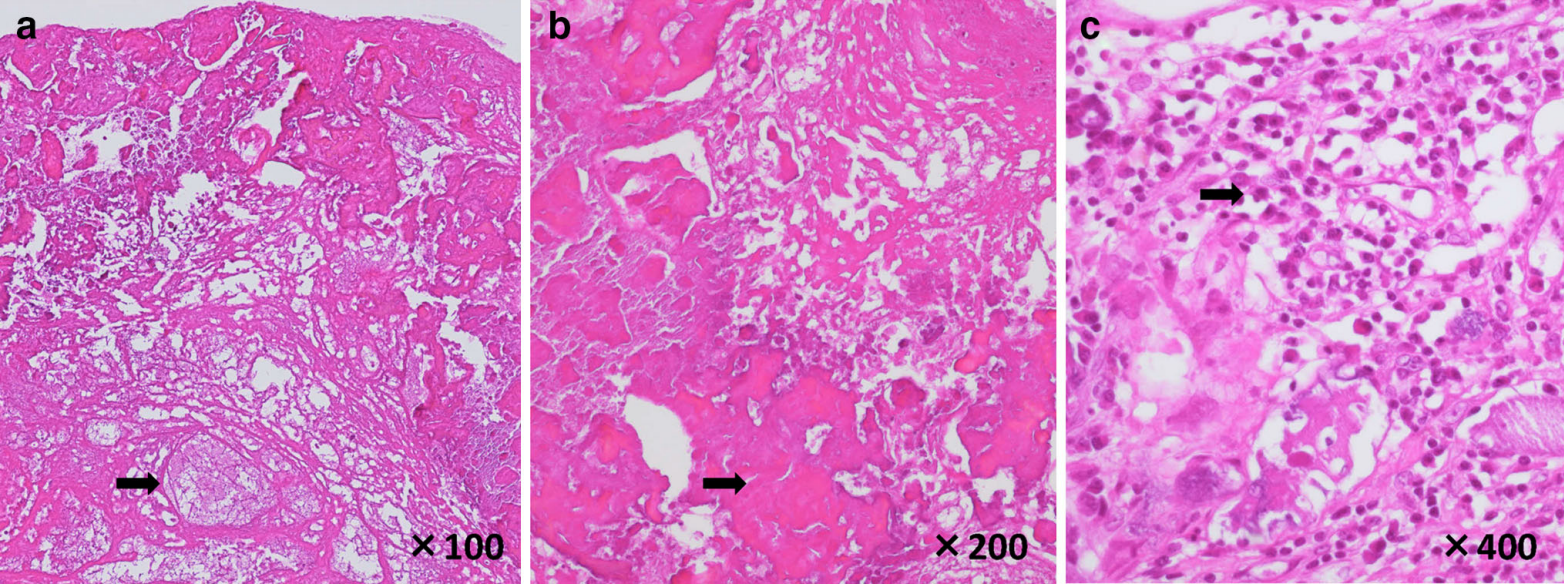
Pathological findings. *Black arrows* indicate calcified nodules (**a**), fibrin (**b**), and lymphocyte and plasma cells (**c**)

A 70-year-old female had received hemodialysis for 23 years because of chronic glomerulonephritis and had undergone implantation of a dual-chamber permanent pacemaker 2 years previously because of a complete atrioventricular block. The patient presented to our hospital with exertional dyspnea and lower limb edema and was diagnosed with acute heart failure. Her body temperature was 36.3 °C, and she had a C-reactive protein concentration of 6.45 mg/dl and brain natriuretic peptide concentration of 916 pg/ml. A repeat blood culture yielded negative results. Chest radiography showed cardiomegaly and pulmonary congestion. Transthoracic echocardiography (TTE) indicated mitral annulus calcification (MAC) and severe mitral regurgitation (MR) (Fig. 1a) due to degeneration as well as moderate tricuspid regurgitation (TR). TTE showed that the MR volume and MR regurgitant fraction were 70 ml and 60 %, respectively. The TR pressure gradient was 47 mmHg. Furthermore, the TTE showed two mobile masses attached to the mitral valve and tricuspid valve annulus (Fig. 1b, d). Transesophageal echocardiography (TEE) revealed a mobile, highly echoic mass on the A1-anterior commissure and septal annulus of the tricuspid valve (Fig. 1c, e). We suspected the presence of a cardiac tumor or vegetation. The patient received 3 g/day sulbactam-ampicillin and 60 mg/day gentamicin. Surgery was performed on the 14th day after hospital admission. She underwent MVR, TAP, tumor resection, and extraction of the permanent pacing leads because infective endocarditis could not be completely excluded. MVR was selected because the anterior leaflets were resected during the tumor resection. The tumor had been attached to the A1-anterior commissure and septal annulus of the tricuspid valve and infiltrated the right ventricular septum. The pathological findings showed calcified nodules in a region of amorphous fibrinous and focal chronic inflammation (Fig. 2). We diagnosed the tumor as a CAT. After surgery, the heart failure was controlled, and the patient’s clinical course was uneventful.

## Discussion

CAT was originally described in 1997 by Reynolds and colleagues [1] as a non-neoplastic cardiac mass. Nearly 50 cases of CAT have been reported. However, this is the first report of a CAT being found on both sides of the atrioventricular valve annulus, and the diagnosis was made by histological examination. A patient with CAT usually exhibits end-stage renal failure or has received hemodialysis. Furthermore, cases of MAC-related mobile CATs, which can mimic vegetation, have been reported [2, 3]. It is thought that abnormalities in the calcium-phosphorus metabolism due to renal dysfunction may contribute to the rapid growth of this tumor. In the preoperative differential diagnosis for the tumor, we considered CAT, vegetation, another cardiac tumor, and thrombosis. The findings were atypical for vegetation because the blood culture yielded negative results, and the Duke criteria were not satisfied. However, it was very difficult to differentiate CAT from this. Therefore, histological examination is necessary to achieve a definitive diagnosis. Although the clinical prognosis is usually good, one case of a recurrent cardiac CAT in a young patient has been reported [4]. Therefore, a patient with MAC who has received hemodialysis or experienced end-stage renal failure should be carefully monitored by echocardiography.
